# Structure evolution of chromium-doped boron clusters: toward the formation of endohedral boron cages†

**DOI:** 10.1039/c8ra09143a

**Published:** 2019-01-23

**Authors:** Xuecheng Shao, Xin Qu, Siyu Liu, Lihua Yang, Jinghai Yang, Xiaohui Liu, Xin Zhong, Shuai Sun, G. Vaitheeswaran, Jian Lv

**Affiliations:** State Key Laboratory for Superhard Materials, College of Physics, Jilin University Changchun 130012 China quxin@calypso.cn; College of Materials Science and Engineering, Jilin University Changchun 130012 China; Key Laboratory of Functional Materials Physics and Chemistry of the Ministry of Education, Jilin Normal University Changchun 130103 China ylh@calypso.cn; National Demonstration Center for Experimental Physics Education, Jilin Normal University Siping 136000 China; Network Information Center, Supercomputing Center, University of Science and Technology of China Hefei 230026 China; Engineering Training Center, Institute of Mechanical Science and Engineering, Jilin University Changchun 130012 China; Advanced Center of Research in High Energy Materials (ACRHEM), University of Hyderabad Hyderabad 500046 India

## Abstract

The electron-deficient nature of boron endows isolated boron clusters with a variety of interesting structural and bonding properties that can be further enriched through metal doping. In the current work, we report the structural and electronic properties of a series of chromium-doped boron clusters. The global minimum structures for CrB_*n*_ clusters with an even number of *n* ranging from 8 to 22 are proposed through extensive first-principles swarm-intelligence structure searches. Half-sandwich structures are found to be preferred for CrB_8_, CrB_10_, CrB_12_ and CrB_14_ clusters and to transform to a drum-like structure at CrB_16_ cluster. Endohedral cage structures with the Cr atom located at the center are energetically most favorable for CrB_20_ and CrB_22_ clusters. Notably, the endohedral CrB_20_ cage has a high symmetry of *D*_2d_ and a large HOMO–LUMO gap of 4.38 eV, whose stability is attributed to geometric fit and formation of an 18-electron closed-shell configuration. The current results advance our understanding of the structure and bonding of metal-doped boron clusters.

## Abstract 2

The effect of chromium doping on the structure evolution of small-sized boron clusters.

## Graphical abstract

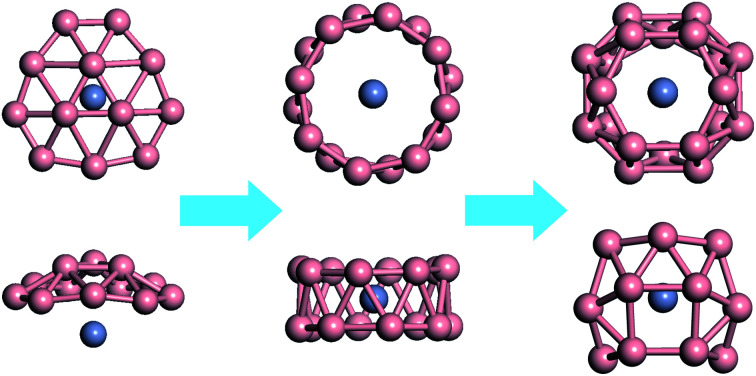

## 1. Introduction

Boron is an element of fascinating structural and chemical complexity, leading to topics of considerable interest in chemistry. It has three valence electrons that are deficient compared with the four valence orbitals, and it prefers to share rather than donate the valence electrons. These characteristics make it difficult for B to achieve filled octets through classical 2c–2e bonds, giving rise to a rich variety of structures along with electron-deficient multicentered bonds, both in its elemental form and chemical compounds.^1^ Sixteen polymorphs have been discovered for bulk B with B_12_ icosahedron being a prevalent motif.^2^ 1D nanotubes^3–5^ and 2D sheets^6–8^ have been fabricated in which triangular planar B lattices with hexagonal holes are found to be energetically favorable.^9^

For 0D B clusters, the situation is even more interesting. Joint photoelectron spectroscopy and theoretical studies carried out over the past decades show that anionic B_*n*_^−^ clusters up to *n* = 38 are planar or quasi-planar,^10,11^ in which delocalized multicentered bonds are responsible for the stabilities.^12^ Theoretical calculations suggested a planar-to-tubular structure transition taking place at B_20_ for neutrals.^13^ Subsequently, combined collision cross section measurements and theoretical calculations confirmed the existence of tubular structures for cationic B_*n*_^+^ clusters with *n* = 16–25.^14^ Most strikingly, the long-sought B fullerene analogue (borospherenes) was first observed at B_40_ (ref. 15) after extensive theoretical investigations,^16–19^ and a series of axially chiral borospherenes were subsequently identified at B_39_^−^,^20^ B_41_^+^ and B_42_^2+^.^21^ For larger B clusters, recent theoretical studies have suggested quasi-planar,^22,23^ tubular,^24^ cage-like^25^ and bilayered^23^ structures as ground states at certain sizes, and core–shell structures are generally expected to be energetically most favorable for *n* > ∼68.^26–28^

The structural diversity of B clusters can be further enriched through metal doping. Metal-centered monocyclic B rings can be formed by transition metal doping of small B_8–10_ anionic clusters^29^ in which NbB_10_^−^ (ref. 30) and TaB_10_^−^ (ref. 30) hold the highest coordination number of 10 in planar molecular species. Interesting half-sandwich structures have been found in Co/Rh doped B_12_ anionic clusters,^31^ and drum-like structures have been observed in CoB_16_^−^,^32^ MnB_16_^−^ (ref. 33) and TaB_20_^−^ (ref. 34) clusters, in which the TaB_20_^−^ cluster possesses the highest coordination number (20) heretofore known in chemistry. Motivated by the discovery of borospherenes in our previous work, we have designed symmetric endohedral B cages, such as MnB_20_^+^,^35^ MoB_24_ (ref. 36) and WB_24_,^36^ whose stabilities are attributed to the formation of 18-electron closed-shell configurations, as well as geometric fits between the sizes of the transition metal atoms and the cavities of the B cages. It was further suggested that, by reasonable choice of the transition metal atom and number of B atoms, other-sized boron cages are also likely to be stabilized. Recently, a theoretical study found that even the small B_12_ cluster can show interesting transitions from quasi-planar to tubular and cage-like structures through interactions with lithium atoms, in which charge transfer plays a critical role.^37^

Metal doping has been proven to be an effective avenue to achieve intriguing structure motifs in B clusters. Given the large number of possible combinations between metal atoms and the number of B atoms yet to be investigated, it not unreasonable to expect more fascinating phenomena in this group of chemical species. As such, in this paper, we report systematic investigations on the structure and bonding of a series of chromium (Cr) doped B clusters by means of the swarm-intelligent CALYPSO structure searching method and first-principles density functional calculations. Ground-state structures are proposed for CrB_*n*_ clusters with an even number of *n* ranging from 8 to 22, revealing an intriguing transition from half-sandwich to drum-like and then endohedral cage-like structures. In particular, a symmetric *D*_2h_ endohedral cage is revealed as the ground-state structure for the CrB_20_ cluster. The rest of the manuscript is organized as follow. The second section describes the computational details. Section 3 presents the results and discussion, and the conclusions from the present results are given in Section 4.

## 2. Calculation details

The unbiased structure searches of CrB_*n*_ clusters with an even number of *n* ranging from 8 to 22 are based on the global minimization of the potential energy surfaces, merging *ab initio* total energy calculations *via* the CALYPSO (Crystal structure AnaLYsis by Particle Swarm Optimization) package.^38–41^ Several major techniques are included in the algorithm to achieve high efficiency, *e.g.*, point group symmetry constraints in structural generation, bond characterization matrix technique for fingerprinting structures, and a local version of the particle swarm optimization algorithm enabling simultaneous search in different energy funnels.^39^ Its validity has been manifested by successful identification of the ground-state structures for a large number of systems. More than 2000 trial structures were generated for each cluster.

During the structure searches, the underlying energy calculations and structure relaxations are carried out in the framework of density functional theory (DFT) with the PBE functional^42^ implemented in the ABACUS (Atomic-orbital Based Ab initio Computation at USTC) package.^43^ ABACUS was developed to perform large-scale DFT simulations using linear combinations of atomic orbitals.^44,45^ The recently developed systematically improvable optimized numerical atomic orbitals^44,45^ were found to be an excellent choice to describe various materials, such as molecules, crystalline solids, surfaces, and defects.^43^ The atomic orbitals basis set of B includes two s, two p and one polarized d orbitals (2s2p1d), whereas the basis set of Cr includes four s, two p, two d and one polarized f orbitals (4s2p2d1f). The radii of the numerical atomic orbitals are set to 7 bohr for B and 8 bohr for Cr in the first-round local structure optimization, while 8 bohr for B and 9 bohr for Cr are used in the second-round local structure optimization. We adopt the SG15 Optimized Norm-Conserving Vanderbilt pseudopotentials,^46^ and the energy cutoff for charge density is 240 Ry.

The low-lying isomers obtained from the structure searches were reoptimized at different spin states at PBE0/Cr/Stuttgart/B/6-311+G* level of theory using the Gaussian 09 Package.^47^ The calculation of harmonic vibrational frequencies ensures that the cluster geometries are true local minima on the potential energy surface (no imaginary frequencies obtained). At this step, singlet, triplet, quintet and septet states were considered for all of these even-number-electrons clusters. In a previous benchmark calculation, the PBE0 functional was confirmed to be suitable for describing the energy difference of isomers of medium-sized boron clusters (*e.g.*, B_20_) compared to the high-level CCSD(T) results.^28^ The natural bond orbital (NBO) and adaptive natural density partitioning (AdNDP) analytical methods were carried out with the Multiwfn package^48^ in order to achieve a better understanding of the bonding mechanism.

## 3. Results and discussion

**Fig. 1:**
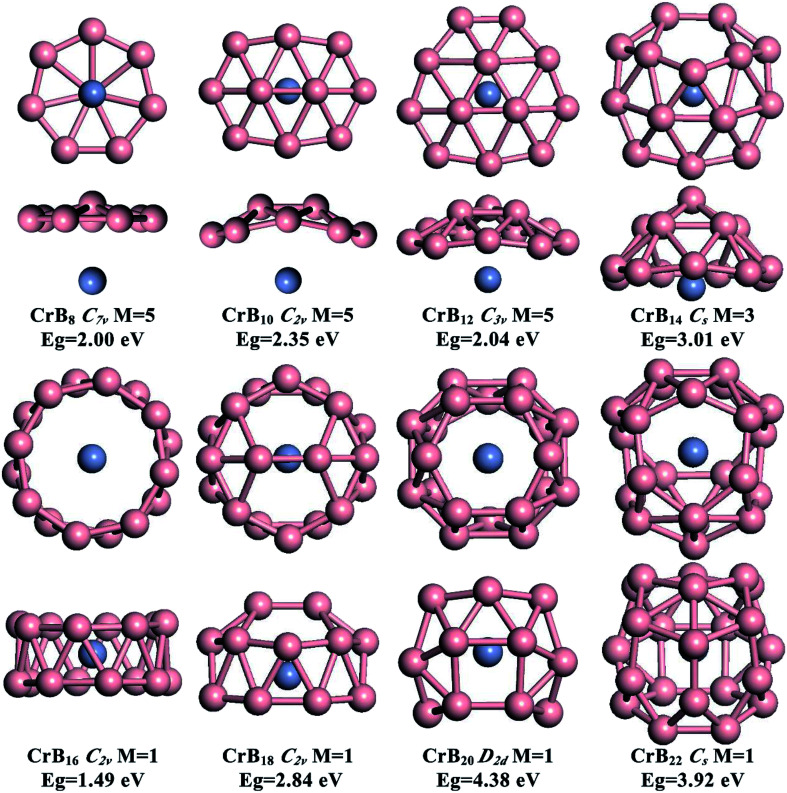
The lowest-energy ground-state isomers of CrB_*n*_ (*n* = 8, 10, 12, 14, 16, 18, 20 and 22) clusters derived from the global minimum structure search. For each system, two sides of the views are given. For each structure, the point group symmetry, spin multiplicity (*M*) and LUMO–HOMO gap (*E*_g_) are indicated.

The global minimum structures for CrB_*n*_ (*n* = 8, 10, 12, 14, 16, 18, 20 and 22) clusters obtained from the current structure searches are depicted in Fig. 1. To facilitate understanding of the structures *via* visualization, top and side views are given along with their point group symmetries, spin multiplicity values (*M*) and HOMO–LUMO energy gaps (*E*_g_). Other low-lying isomers are shown in the ESI, Fig. S1–S8.† Generally, the effect of Cr doping on the structures of B clusters gradually is enhanced as the number of B atoms increases. For small-sized CrB_*n*_ (*n* = 8, 10 and 12) clusters, the B structures are similar to those in bare B clusters, while larger CrB_*n*_ clusters exhibit B structures different from the corresponding bare B clusters. This leads to interesting transitions from half-sandwich to drum-like and then to endohedral cage-like structures as the number of B atoms increases. High spin states (triplet and quintet states) are preferred for small-sized CrB_*n*_ clusters with *n* < 14, and the magnetism is completely quenched for CrB_*n*_ clusters with *n* ≥ 16.

### 3.1 CrB_8_, CrB_10_, CrB_12_ and CrB_14_ clusters with half-sandwich structures

As depicted in Fig. 1, small-sized CrB_*n*_ clusters with *n* = 8, 10, 12 and 14 exhibit half-sandwich structures, whereas quasi-planar or bowl-like B_*n*_ moieties are coordinated to the Cr atom. High spin states are found to be the ground states for these global-minimum structures (quintets for CrB_8_, CrB_10_, CrB_12_, and triplets for CrB_14_). Other low-lying isomers are given in Fig. S1–S8 in the ESI.† Spin density distributions shown in ESI, Fig. S9† indicates that the magnetism mainly originates from the unpaired 3d electrons of the Cr atom.

The B structures in CrB_8_, CrB_10_ and CrB_12_ are very similar to those in bare B clusters, which are quasi-planar with one, two and three interior B atoms surrounded by seven-, eight- and nine-membered B rings, respectively. Due to the existence of the Cr atom, the interior B atoms in the B moieties display slight out-of-plane distortions. Note that, although CrB_10_ is isovalent to NbB_10_^−^ and TaB_10_^−^, it does not adopt the metal-centered monocyclic B rings in NbB_10_^−^ and TaB_10_^−^ as the ground state. This may be due to the smaller size of the Cr atom (1.39 Å) compared with those of Nb (1.64 Å) and Ta (1.70 Å), which is not optimal for fitting the cavity of the ten-membered B ring. The half-sandwich structure of CrB_12_ is the same as those in experimental CoB_12_^−^ and RhB_12_^−^, further indicating that the double aromatic B_12_ moiety is a promising inorganic ligand.

Inserting two B atoms into the B_12_ moiety in the CrB_12_ cluster leads to the formation of CrB_14_. The B_14_ moiety in CrB_14_ has a bowl-like structure with five interior B atoms surrounded by a nine-membered B ring, which is different from the structures of neutral or charged bare B_14_ clusters.^10,49^ In contrast to CrB_8_, CrB_10_ and CrB_12_, the Cr atom is half encircled by the bowl-like B_14_ moiety in CrB_14_, leading to enhanced Cr–B interactions and partially quenched magnetism. The significant curving of the B moiety in CrB_14_ indicates the gradually enhanced effect of Cr doping on the structure evolution and onset of structure transition in CrB_*n*_ clusters.

### 3.2 The CrB_16_ cluster with a drum-like structure

The well-known drum-like structure with the Cr atom located at the center of a B_16_ double-ring tube occurs with the CrB_16_ cluster, having a point group of *C*_2v_ (Fig. 1). Within this structure, the magnetism is completely quenched due to the strong coordination interactions between the Cr atom and the B_16_ tube. This type of structure was initially observed in CoB_16_^−^ (ref. 32) and MnB_16_^−^ (ref. 33) by joint photoelectron spectroscopy measurement and *ab initio* calculations. Later, studies found that larger B_18_ and B_20_ tubes can be stabilized by doping larger transition metal atoms (Rh and Ta), leading to drum-like RhB_18_^−^ (ref. 50) and TaB_20_^−^,^34^ respectively. Interestingly, recent theoretical studies have found that the smaller B_14_ tube can be formed by the doping of an Fe atom,^51^ though the size of Fe (1.32 Å) is larger than that of Co (1.26 Å). Thus, it seems that whether drum-like structures can be formed in metal-doped B clusters is closely related to the size of the doping atom and the cavity of the B tube, as well as the charge and spin states of the metal-doped B clusters.

**Fig. 2:**
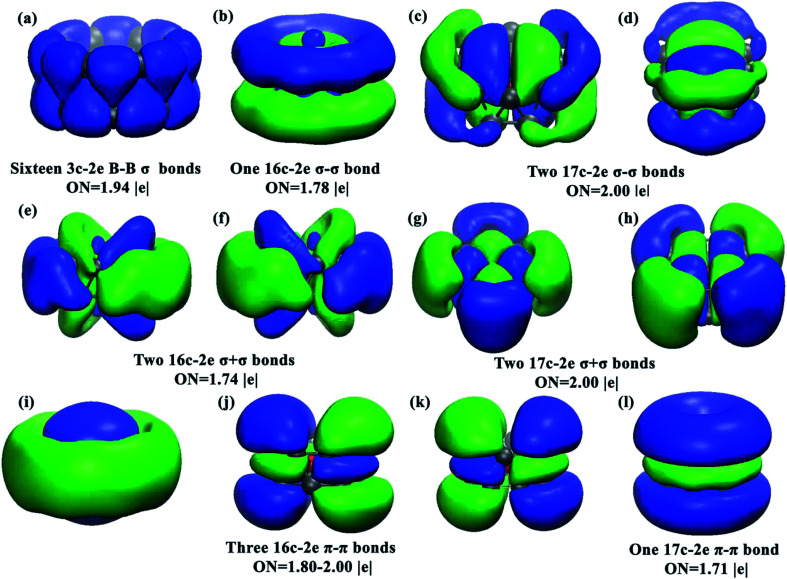
Images of the chemical bonding of CrB_16_ obtained from AdNDP analyses. ON stands for occupation number.

The chemical bonding of the current drum-like CrB_16_ cluster was analyzed using the Adaptive Natural Density Partitioning (AdNDP)^52^ method, which is an extension of the Natural Bond Orbital method.^53^ AdNDP analyses can display both localized and delocalized bonding in molecules simultaneously, providing relatively simple bonding pictures for complicated molecular structures.^33^ The AdNDP analyses revealed a similar bonding character for CrB_16_ as those of CoB_16_^−^ and MnB_16_^−^, where the 54 valence electrons in CrB_16_ can be divided into four bonding types as shown in Fig. 2. The occupation numbers (ON) of all the identified bonds range from 1.71 to 2.00 |*e*|. The first type represents localized bonds (Fig. 2a), which can be described in two manners: (1) as sixteen 3c–2e σ bonds on the sixteen B_3_ triangles on the drum surface with ON = 1.94 |*e*| or (2) as sixteen 2c–2e σ bonds within the two B_8_ rings with ON = 1.73 |*e*|. In fact, the sixteen 3c–2e σ bonds can also be represented by sixteen 2c–2e σ bonds on the two B_8_ rings on the drum surface are shown in Fig. S10 in ESI.†

The remaining three bonding types describe totally delocalized bonds (Fig. 2b–d, e–i and j–m) and account for bonding between the two B_8_ rings and between the Cr atom and the B_16_ tube. Following the previous work on CoB_16_^−^ and MnB_16_^−^, the “+” sign is used to denote that the delocalized bonds between the two B_8_ rings overlap positively, while the “−” sign means a negative overlap. The second bonding type (Fig. 2b–d) consists of one 16c–2e σ − σ bond with ON = 1.78 |*e*| and two 17c–2e σ − σ bonds with ON = 2.00 |*e*|. The one 16c–2e σ − σ bond represents a bonding interaction within each B_8_ ring and an antibonding interaction between the two B_8_ rings, while the two 17c–2e σ − σ bonds represent mainly covalent bonding between Mn (3d_*xz*_ and 3d_*yz*_) and the B_16_ tube. The third bonding type (Fig. 2e–h) contains two 16c–2e σ + σ bonds with ON = 1.74 |*e*| and two 17c–2e σ + σ bonds with ONs = 2.00 |*e*|. The two 16c–2e bonds represent delocalized σ bonding in the B_16_ frame, and the two 17c–2e σ + σ bonds represent covalent bonding between Cr (3d_*xy*_ and 3d_*x*^2^−*y*^2^_) and the B_16_ tube. The fourth bonding type consists of three 16c–2e π − π bonds and one 17c–2e π − π bond. These four bonds account for π bonding interactions between the two B_8_ rings.

### 3.3 Transition from drum-like to endohedral cage-like structures in CrB_18_, CrB_20_ and CrB_22_ clusters

As depicted in Fig. 1, capping two B atoms on one side of the drum-like CrB_16_ structure leads to the global minimum structure of the CrB_18_ cluster, which can be seen as an intermediate structure between drum-like and endohedral cage-like structures. Notably, a symmetric endohedral cage with the Cr atom located at the center emerges as the global minimum structure for the CrB_20_ cluster. The structure is composed of twenty B triangles, four B quadrangles and two B hexagons in *D*_2d_ symmetry. It has the largest HOMO–LUMO gap of 4.38 eV among CrB_*n*_ clusters considered in the current work. The high symmetry and large HOMO–LUMO gap imply highly degenerate electronic states and the potentially high chemical stability of the CrB_20_ cluster. Further addition of two B atoms into the CrB_20_ cluster also leads to an endohedral cage-like structure in the CrB_22_ cluster, suggesting that endohedral cage-like motifs may be prevalent for larger CrB_*n*_ clusters with *n* > 20. However, the endohedral CrB_22_ cage is rather irregular, and its HOMO–LUMO gap (3.92 eV) is smaller than that of CrB_20._

Early theoretical studies have demonstrated metal doping as a viable route for stabilizing cage-like B structures. A series of transition-metal-centered endohedral B cages have been proposed, such as FeB_18_, FeB_20_, MnB_20_^+^, MoB_24_, and WB_24_. However, the transition metal should possess both geometric and electronic states that can fit in high symmetric endohedral B cage-like clusters that eventually lead to the high stability structures that can be formed. The current endohedral *D*_2d_ CrB_20_ cage should be one such paradigm. The geometric factor responsible for the stability is straightforward. Our previous calculations have demonstrated that the Cr atom is too small to fit a large B_24_ cage, indicating smaller B cages are suitable for accommodating one Cr atom.

**Fig. 3:**
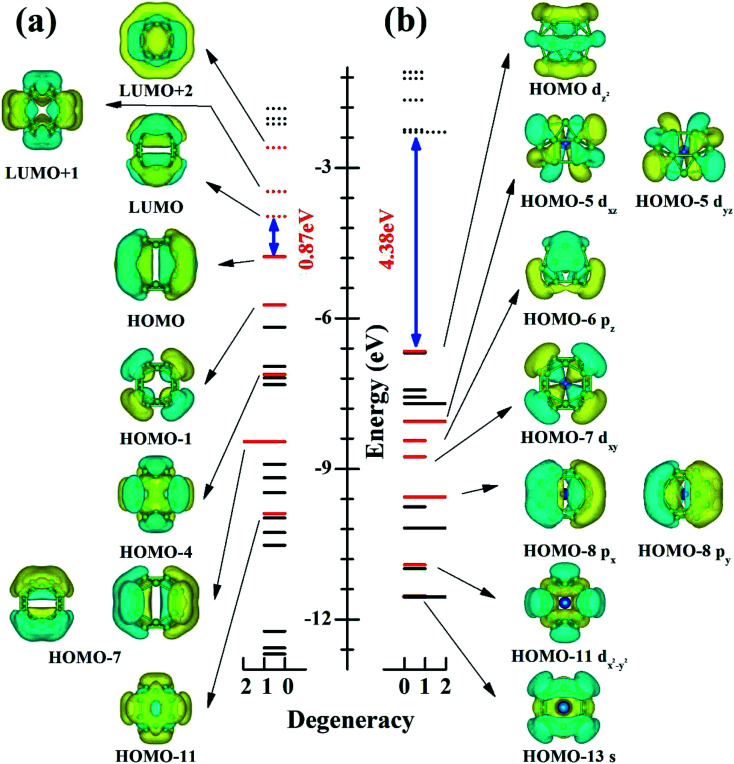
Eigenvalue spectrum *vs.* electronic state degeneracy of (a) bare *D*_2d_–B_20_ cage and (b) *D*_2d_–CrB_20_. For each case, the HOMO–LUMO gap is indicated (in blue). The π-orbitals (red lines) (a) and the orbitals involving the 18-electron closed-shell configuration (b) are shown.

To understand electronic factors responsible for the stability of the endohedral CrB_20_ cage, the chemical bonding of the bare *D*_2d_ B_20_ cage and endohedral *D*_2d_ CrB_20_ cage were analyzed based on canonical molecular orbitals (CMOs). Fig. 3 shows the comparison of eigenvalue spectra for the *D*_2d_ cage without (a) and with (b) Cr encapsulation. One can clearly note that the Cr encapsulation significantly increases the HOMO–LUMO gap from 0.87 eV for the bare *D*_2d_ B_20_ cage to 4.38 eV for the *D*_2d_ CrB_20_. For bare *D*_2d_ B_20_ (Fig. 3a), there are 6 occupied π-orbitals (HOMO, HOMO−1, HOMO−4, two HOMO−7, and HOMO−11) and 3 unoccupied π-orbitals (LUMO, LUMO+1 and LUMO+2). The moderate HOMO–LUMO gap in bare *D*_2d_ B_20_ is attributed to the mid-lying binding energies of these out-of-surface delocalized π-orbitals, and they will interact with the electronic orbitals of Cr atoms in the endohedral CrB_20_ cages. Cr has an electronic configuration of [Ar]4s^1^3d^5^ with 6 valence electrons, adding the 12 π-electrons (from 6 occupied π-orbitals) of bare *D*_2d_ B_20_ gives a total of 18 electrons. This special electron counting number of 18 is favorable for forming a stable 18-electron closed-shell configuration, similar to that of previous MnB_20_^+^, MoB_24_ and WB_24_ clusters. Indeed, nine CMOs involved in the “spd–π interaction” have been identified for the *D*_2d_ CrB_20_ endohedral cage as depicted in Fig. 3b, *i.e.*, HOMO−13 (s-like), HOMO−11 (d_*x*^2^−*y*^2^_-like), HOMO−8 (double degenerate, p_*x*_-like and p_*y*_-like), HOMO−7 (d_*xy*_-like), HOMO−6 (p_*z*_-like), HOMO−5 (double degenerate, d_*xz*_-like and d_*yz*_–π like) and HOMO−1 (3d_*z*^2^_-like). Thus, the CrB_20_ cluster represents another example of having a symmetric endohedral cage configuration stabilized by the 18-electron configuration.

## 4. Conclusions

In summary, we systematically investigated the structural and electronic properties of CrB_*n*_ clusters with *n* = 8, 10, 12, 14, 16, 18, 20 and 22 through extensive swarm-intelligent structure searches and first-principles calculations. It is found that Cr doping significantly modifies the structural evolution of B clusters. Intriguing transitions from half-sandwich to drum-like and then to endohedral cage-like structures are revealed as the number of B atoms increases. CrB_8_, CrB_10_ and CrB_12_ clusters exhibit half-sandwich structure with quasi-planar B moieties similar to the bare B cluster, indicating that small-sized B clusters are promising inorganic ligands. A drum-like structure is formed with CrB_16_ clusters, while endohedral cage structures emerge with the larger CrB_20_ and CrB_22_ clusters. The endohedral CrB_20_ cage has a high symmetry of *D*_2d_ and the largest HOMO–LUMO gap among CrB_*n*_ in the current work, indicating its high chemical stability, which is attributed to the geometric fit between the size of the Cr atom and the void of the B cage as well as the formation of the 18-electron configuration.

## Conflicts of interest

The authors declare no competing financial interest.

## Supplementary Material

RA-009-C8RA09143A-s001
