# Supplementary material for: Structure evolution of chromium-doped boron clusters: toward the formation of endohedral boron cages
Source: RSC Adv. 2019 Jan 23;9(5):2870–6. doi: 10.1039/c8ra09143a (PMC9060308; doi:10.1039/c8ra09143a)
Supplement: RA-009-C8RA09143A-s001 [file RA-009-C8RA09143A-s001.pdf]

Electronic Supplementary Material (ESI) for RSC Advances.
This journal is © The Royal Society of Chemistry 2019

Supporting Information

Structure Evolution of Chromium-Doped Boron Clusters: Toward the Formation of Endohedral Boron Cages

Xuecheng Shao,[1#] Xin Qu,[1,2,3,4#*] Siyu Liu,[1] Lihua Yang,[3,4] Jinghai Yang[3,4] Xiaohui Liu,[5] Xin Zhong,[3,4*] Shuai Sun,[6] G. Vaitheeswaran,[7] and Jian Lv[1,2*]

[1]*State Key Laboratory for Superhard Materials, College of Physics, Jilin University, Changchun, 130012, China*

[2]*College of Materials Science and Engineering, Jilin University, Changchun 130012, China*

[3]*Key Laboratory of Functional Materials Physics and Chemistry of the Ministry of Education, Jilin Normal University, Changchun 130103, China*

[4]*National Demonstration Center for Experimental Physics Education, Jilin Normal University, Siping 136000, China*

[5]*Network Information Center, Supercomputing Center, University of Science and Technology of China, Hefei 230026, China*

[6]*Engineering Training Center,Institute of Mechanical Science and Engineering,Jilin University ,Changchun,130012, China*

[7]*Advanced Center of Research in High Energy Materials (ACRHEM), University of Hyderabad, Hyderabad, 500046, India*

#*Equally share the first authorship*

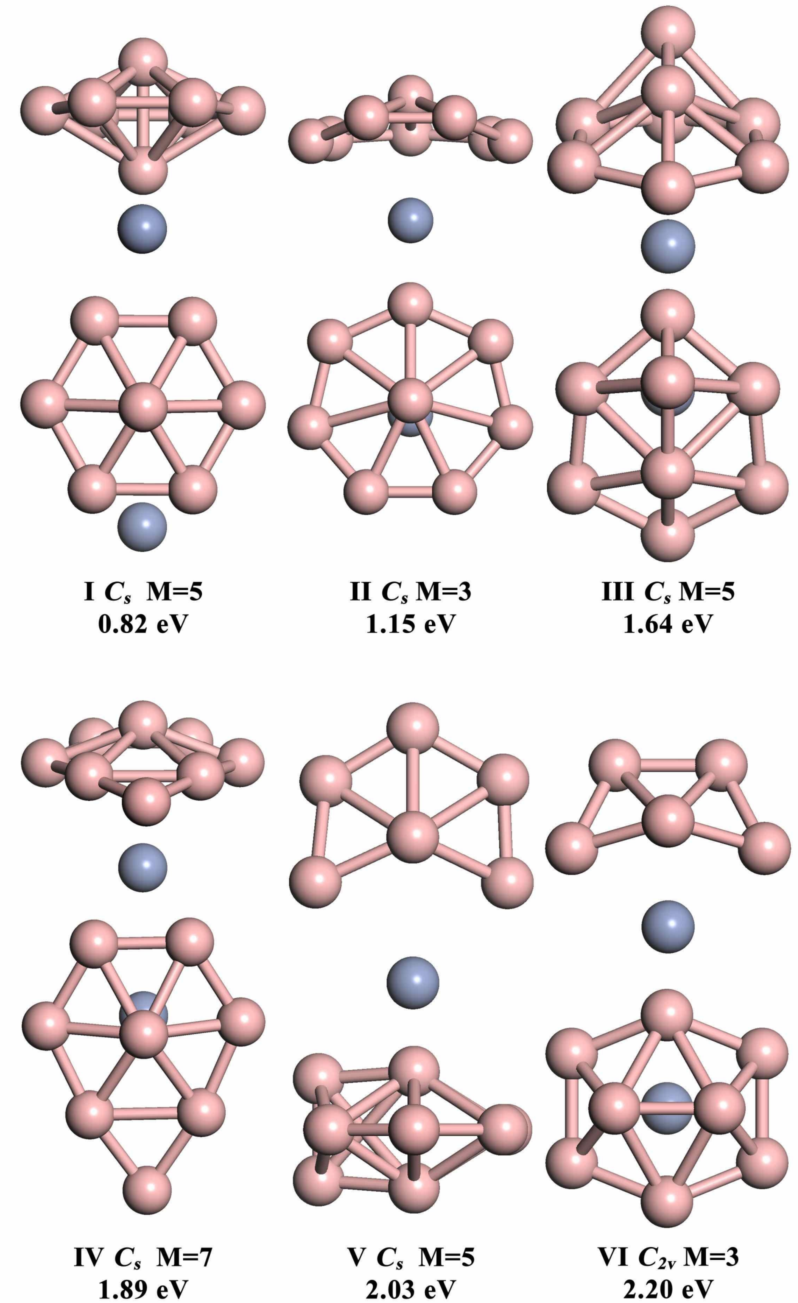

Fig. S1. Structures of low-lying isomers of CrB_8. Bottom labels are point-group symmetries, spin multiplicities and relative energies to the ground state at PBE0/Cr/Stuttgart/B/6-311G* level of theory. Zero-point energy corrections were included for all isomers at the same level of theory.

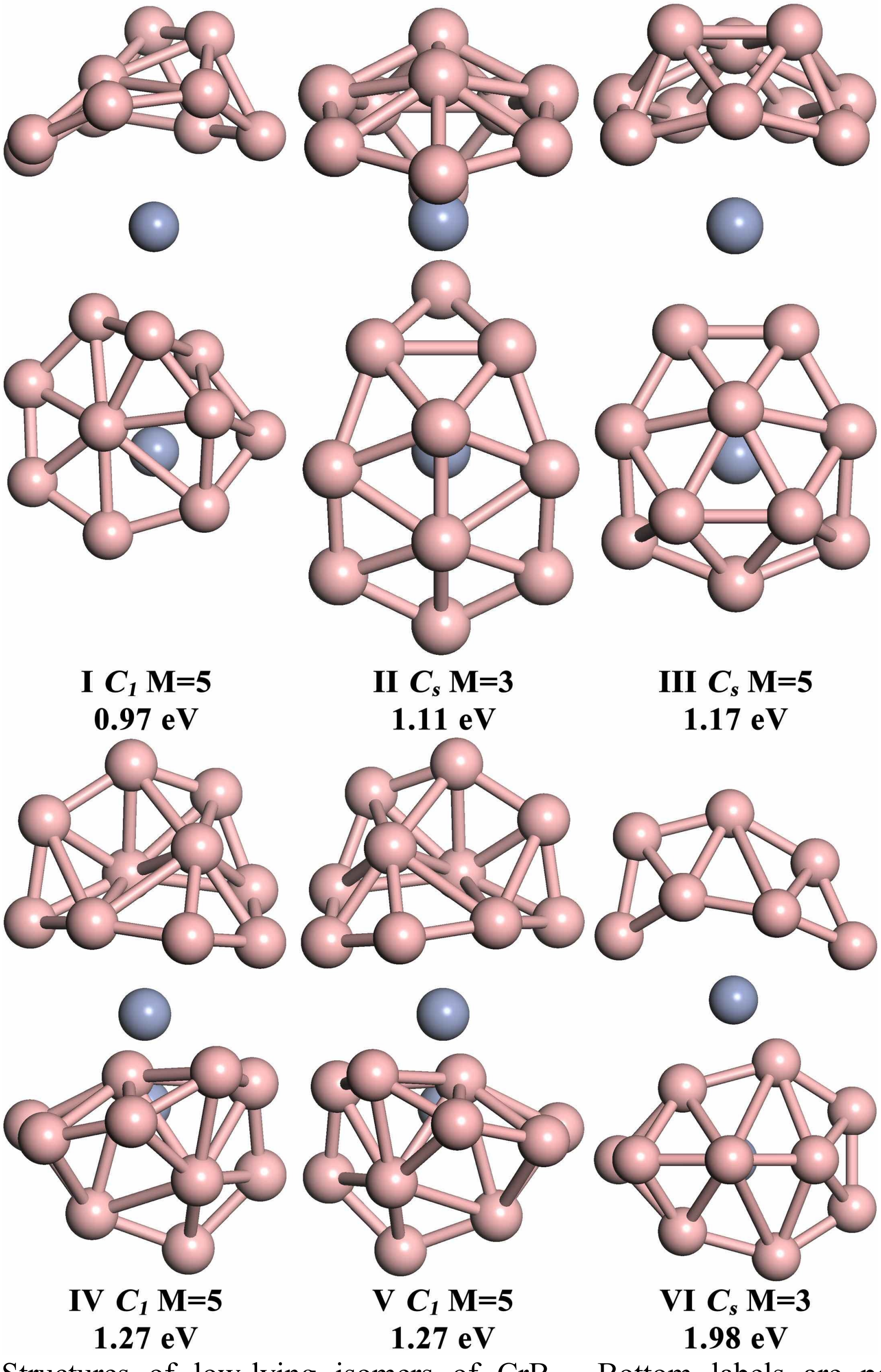

Fig. S2. Structures of low-lying isomers of CrB_{10}. Bottom labels are point-group symmetries, spin multiplicities and relative energies to the ground state at PBE0/Cr/Stuttgart/B/6-311G* level of theory. Zero-point energy corrections were included for all isomers at the same level of theory.

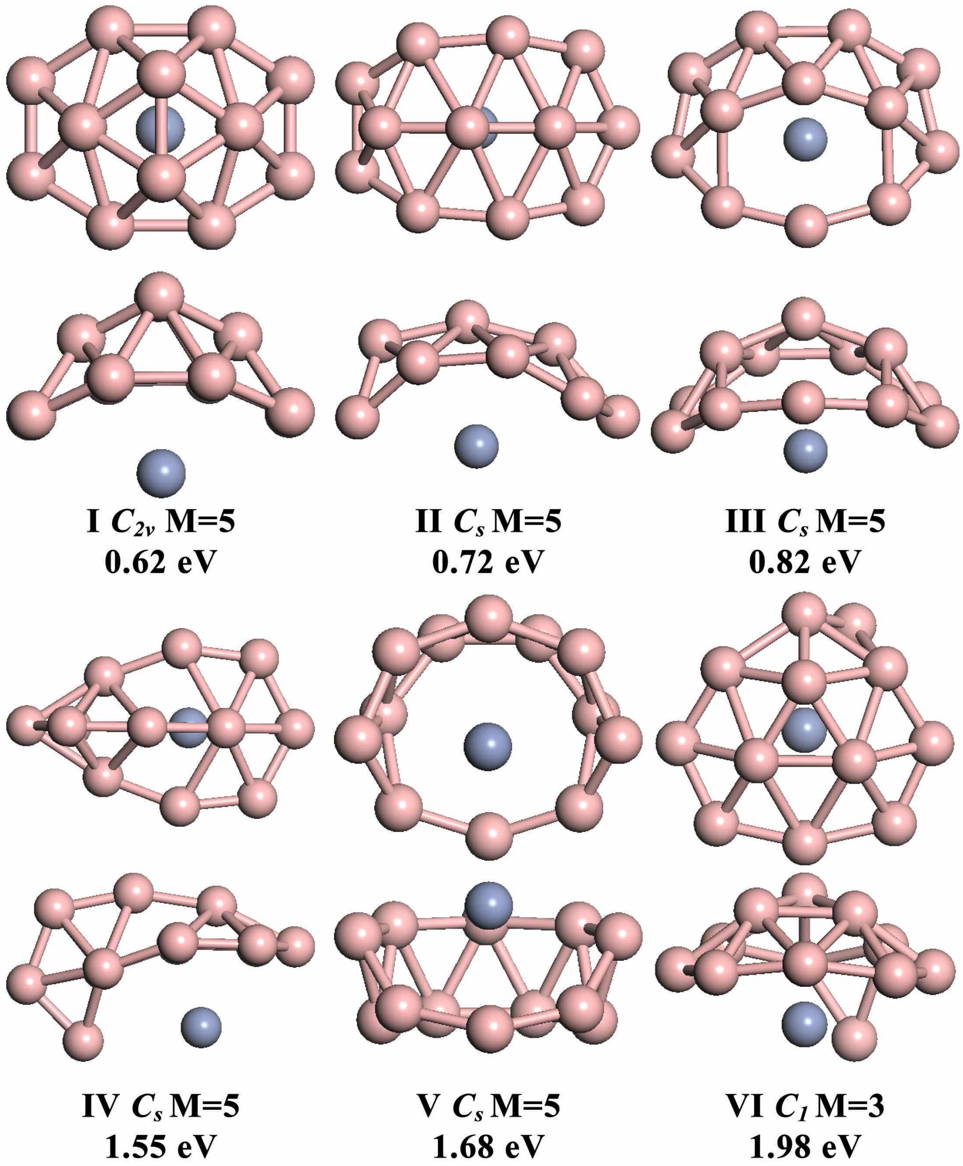

Fig. S3. Structures of low-lying isomers of CrB_{12}. Bottom labels are point-group symmetries, spin multiplicities and relative energies to the ground state at PBE0/Cr/Stuttgart/B/6-311G* level of theory. Zero-point energy corrections were included for all isomers at the same level of theory.

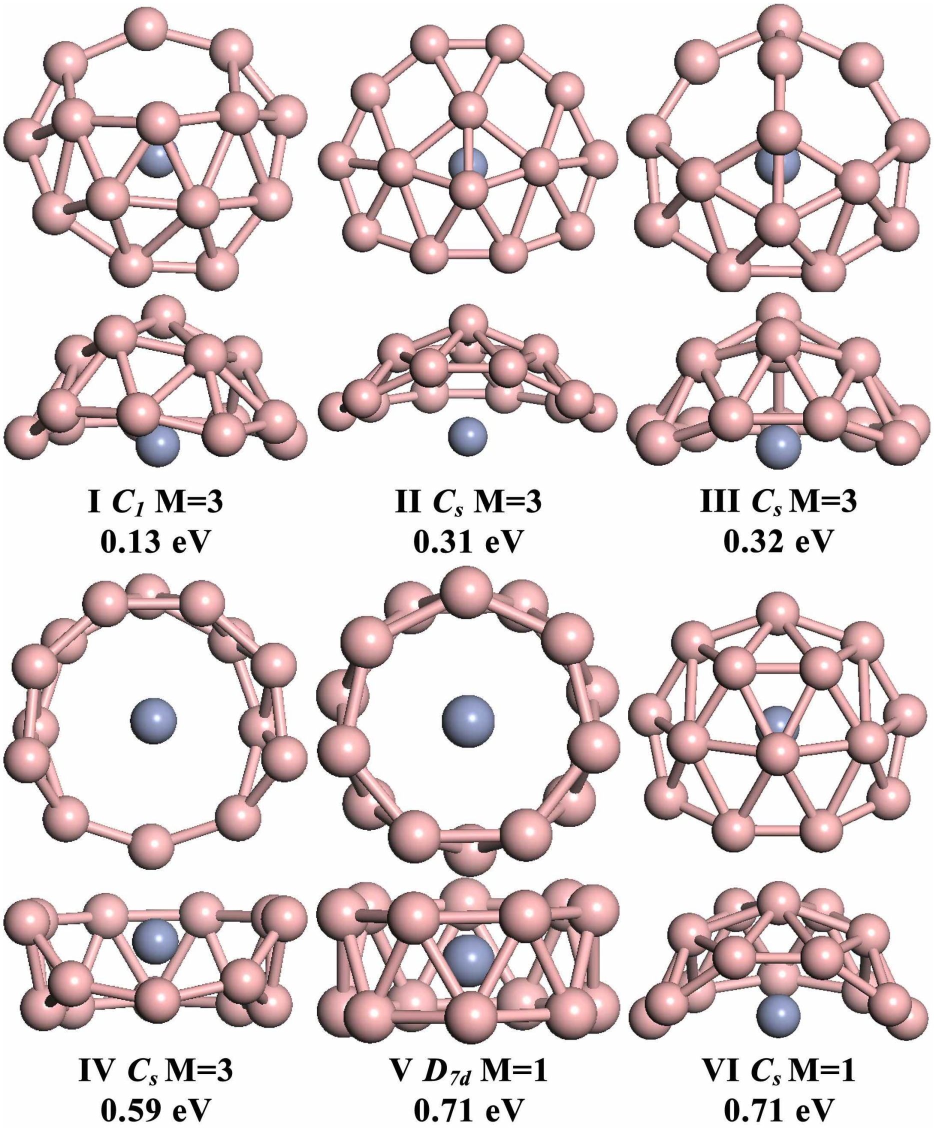

Fig. S4. Structures of low-lying isomers of CrB_{14}. Bottom labels are point-group symmetries, spin multiplicities and relative energies to the ground state at PBE0/Cr/Stuttgart/B/6-311G* level of theory. Zero-point energy corrections were included for all isomers at the same level of theory.

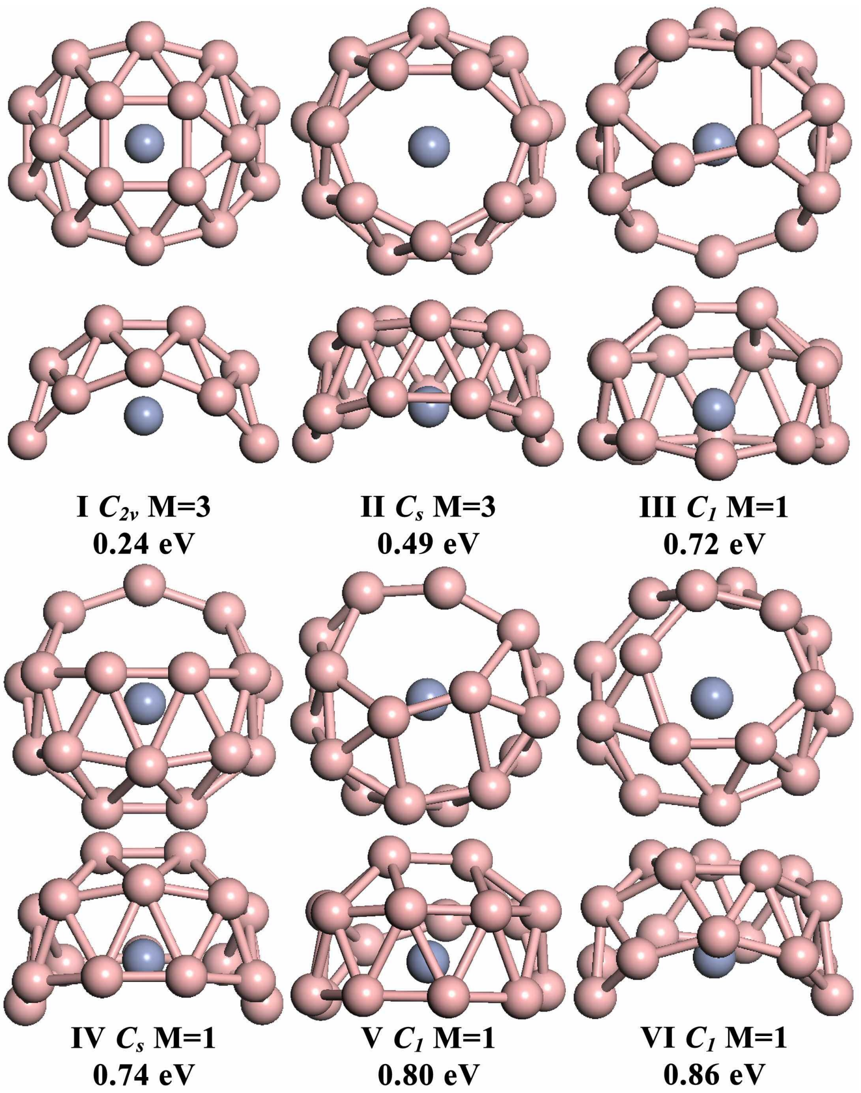

Fig. S5. Structures of low-lying isomers of CrB_{16}. Bottom labels are point-group symmetries, spin multiplicities and relative energies to the ground state at PBE0/Cr/Stuttgart/B/6-311G* level of theory. Zero-point energy corrections were included for all isomers at the same level of theory.

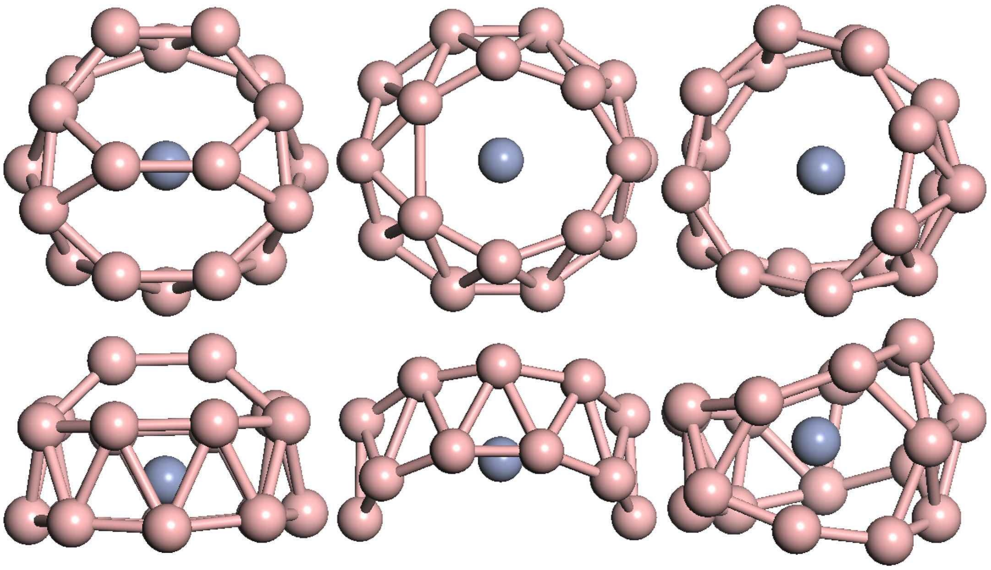

I C_s M=1 **II C_s M=3** **III C_1 M=3**

0.05 eV **1.12 eV** **1.23 eV**

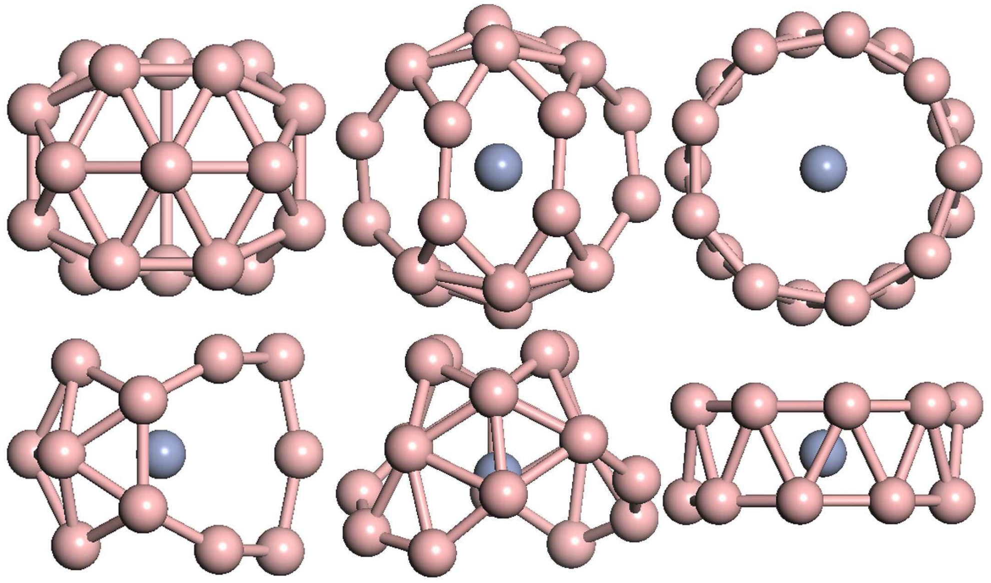

IV C_{2v} M=1 **V C_2 M=3** **VI D_{9d} M=5**

1.36 eV **1.38 eV** **1.44 eV**

Fig. S6. Structures of low-lying isomers of CrB_{18}. Bottom labels are point-group symmetries, spin multiplicities and relative energies to the ground state at PBE0/Cr/Stuttgart/B/6-311G* level of theory. Zero-point energy corrections were included for all isomers at the same level of theory.

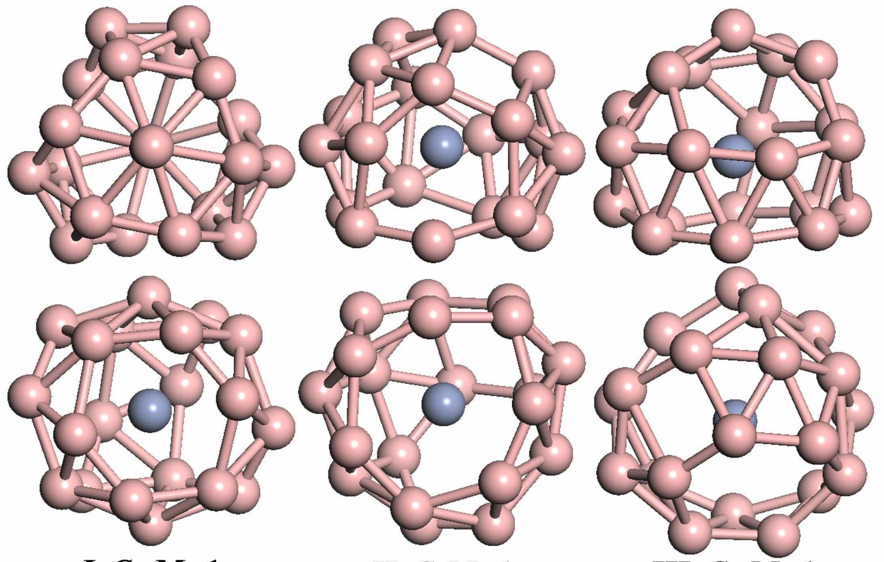

I *C₃* M=1 **II *C₁* M=1** **III *C₁* M=1**

0.32 eV **0.34 eV** **0.39 eV**

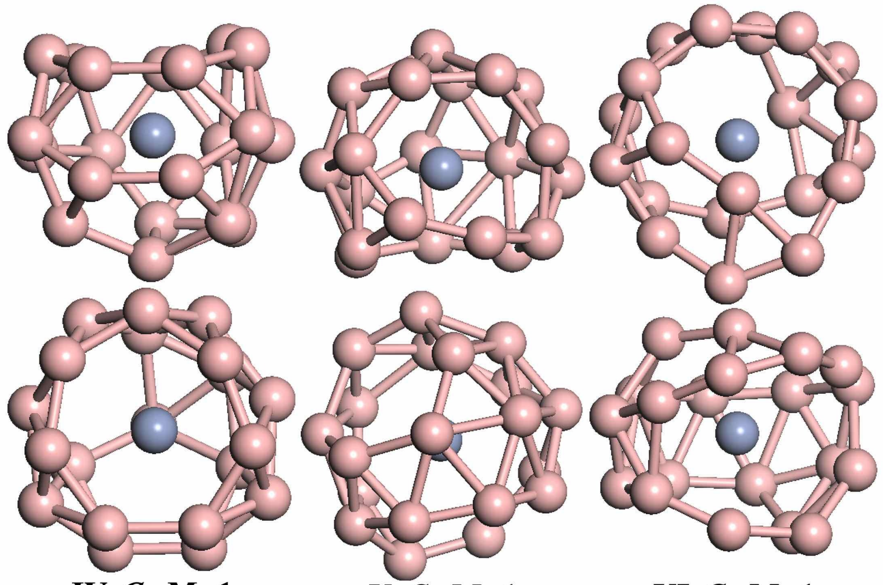

IV *C₁* M=1 **V *C₁* M=1** **VI *C₁* M=1**

0.41 eV **0.50 eV** **1.94 eV**

Fig. S7. Structures of low-lying isomers of CrB_{20}. Bottom labels are point-group symmetries, spin multiplicities and relative energies to the ground state at PBE0/Cr/Stuttgart/B/6-311G* level of theory. Zero-point energy corrections were included for all isomers at the same level of theory.

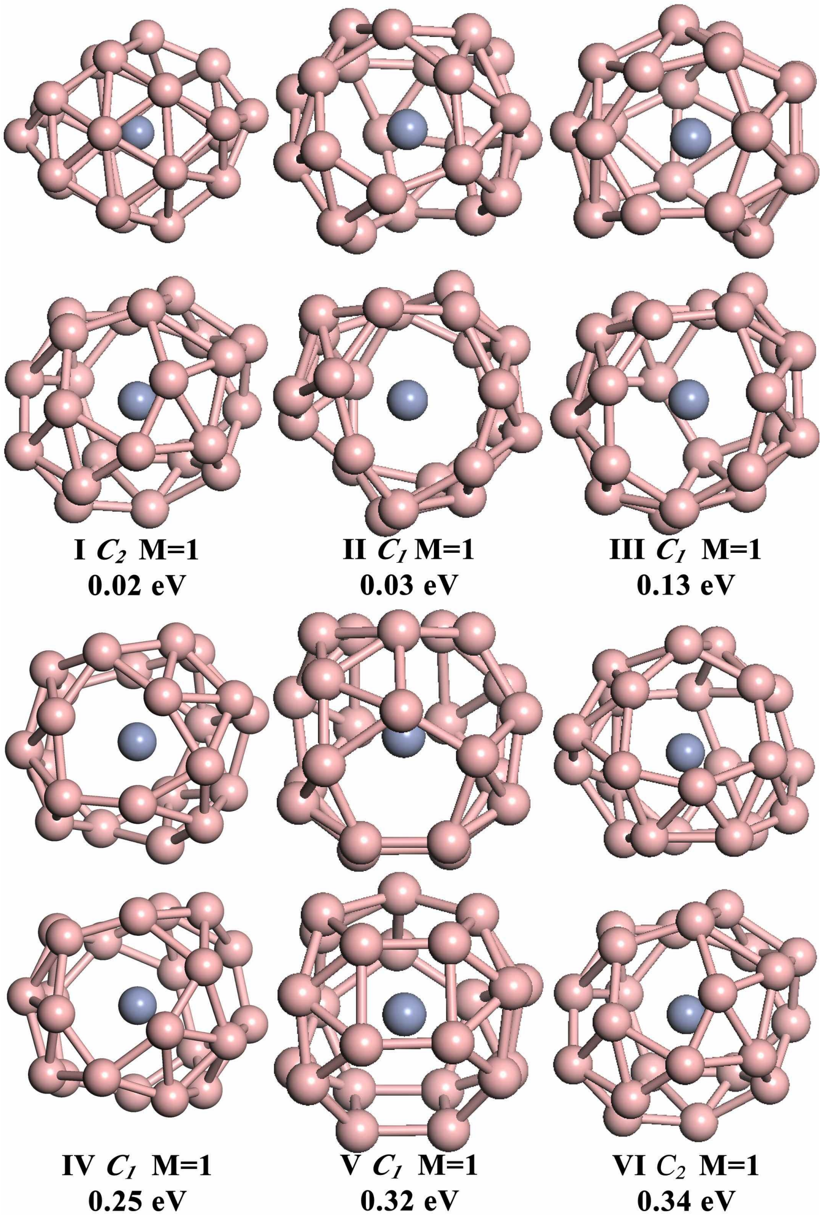

Fig. S8. Structures of low-lying isomers of CrB_{22}. Bottom labels are point-group symmetries, spin multiplicities and relative energies to the ground state at PBE0/Cr/Stuttgart/B/6-311G* level of theory. Zero-point energy corrections were included for all isomers at the same level of theory.

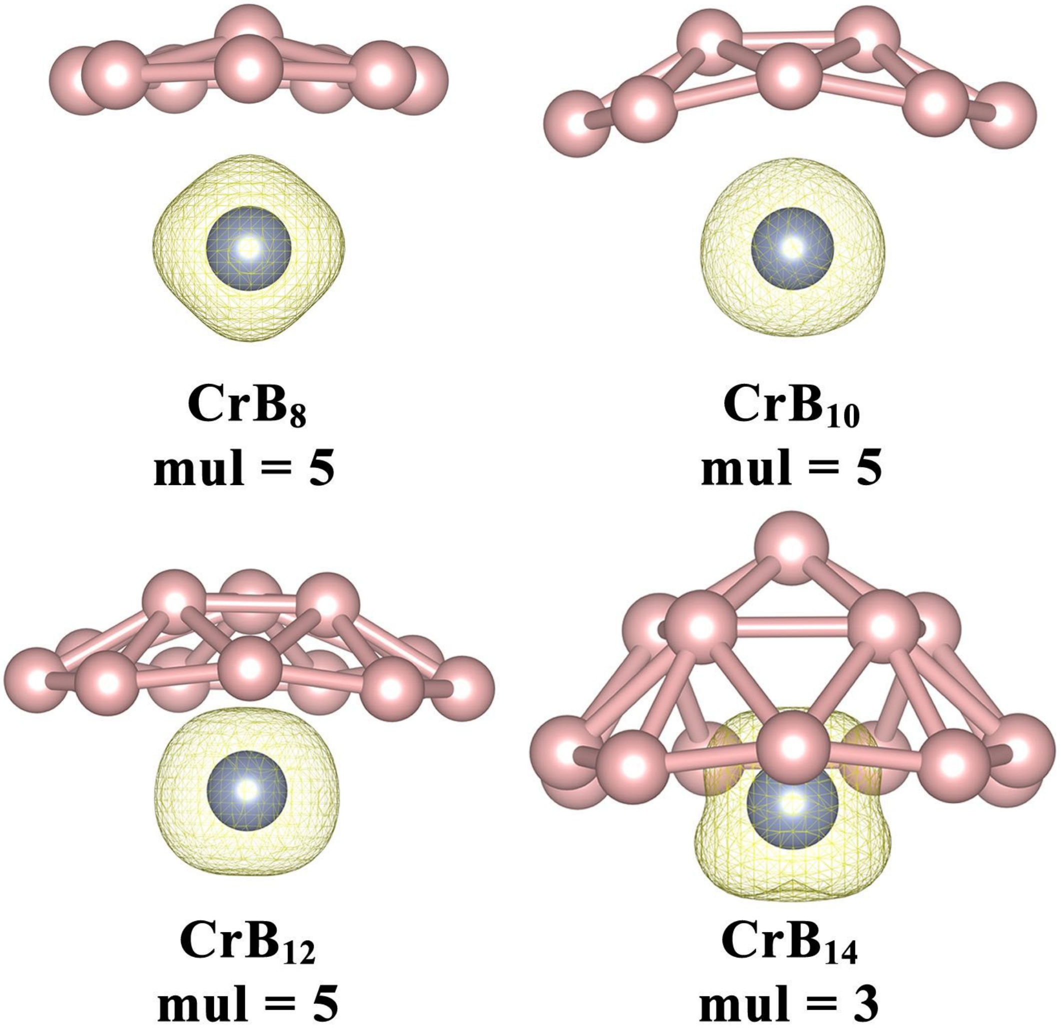

Fig. S9. Electron spin density of CrB_{12}, CrB_{14} and CrB_{16} shows that with the addition of boron atoms, the magnetism of Chromium is vanished. The mul represent the charge spin multiplicity.

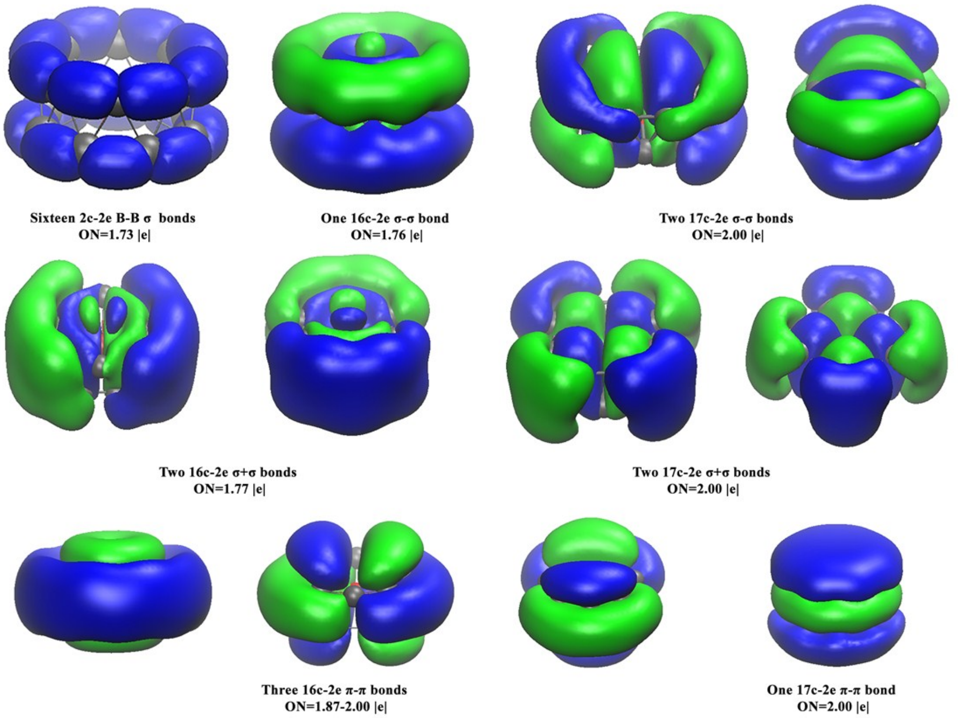

Fig. S10. The chemical bonding picture of CrB_{16} obtained from AdNDP analyses. ON stands for occupation number. Residual valence electrons of all atoms in the search list: 5.17.

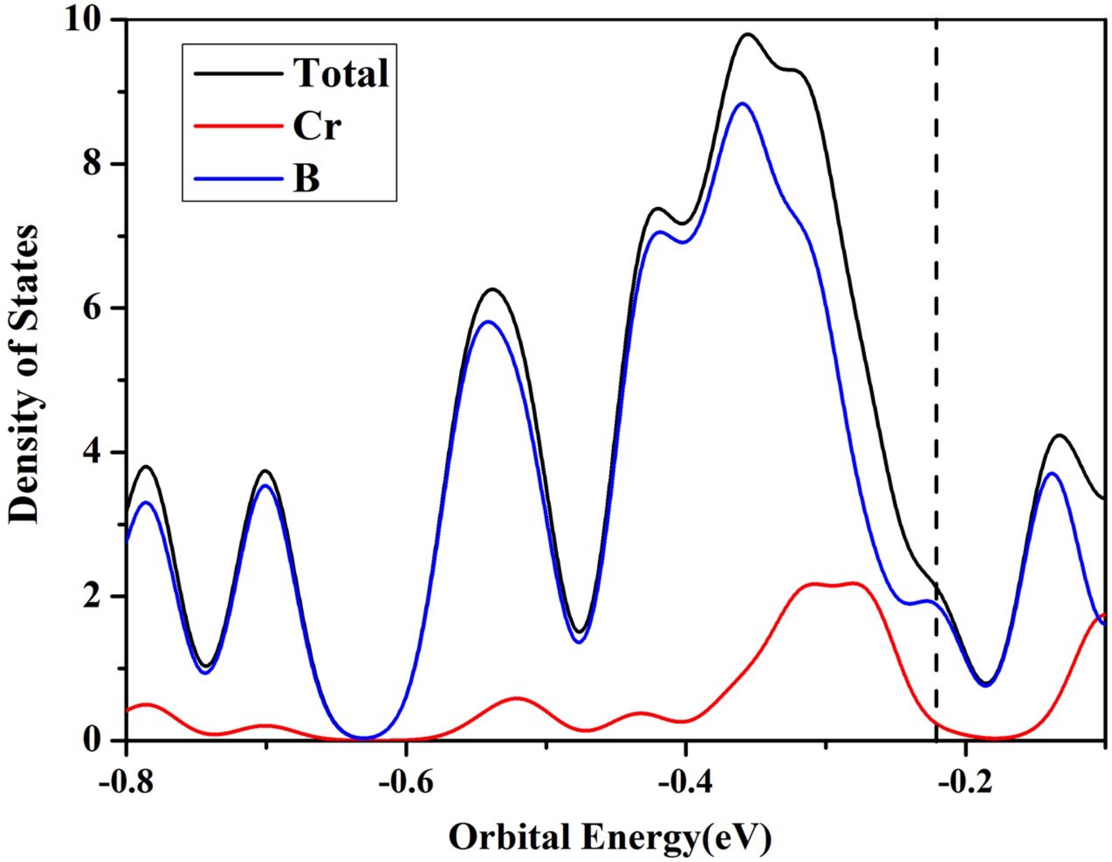

Fig. S11. Total and projected density of states for the double-ring tubular CrB_{16} cluster. The dashed line highlights the position of HOMO.

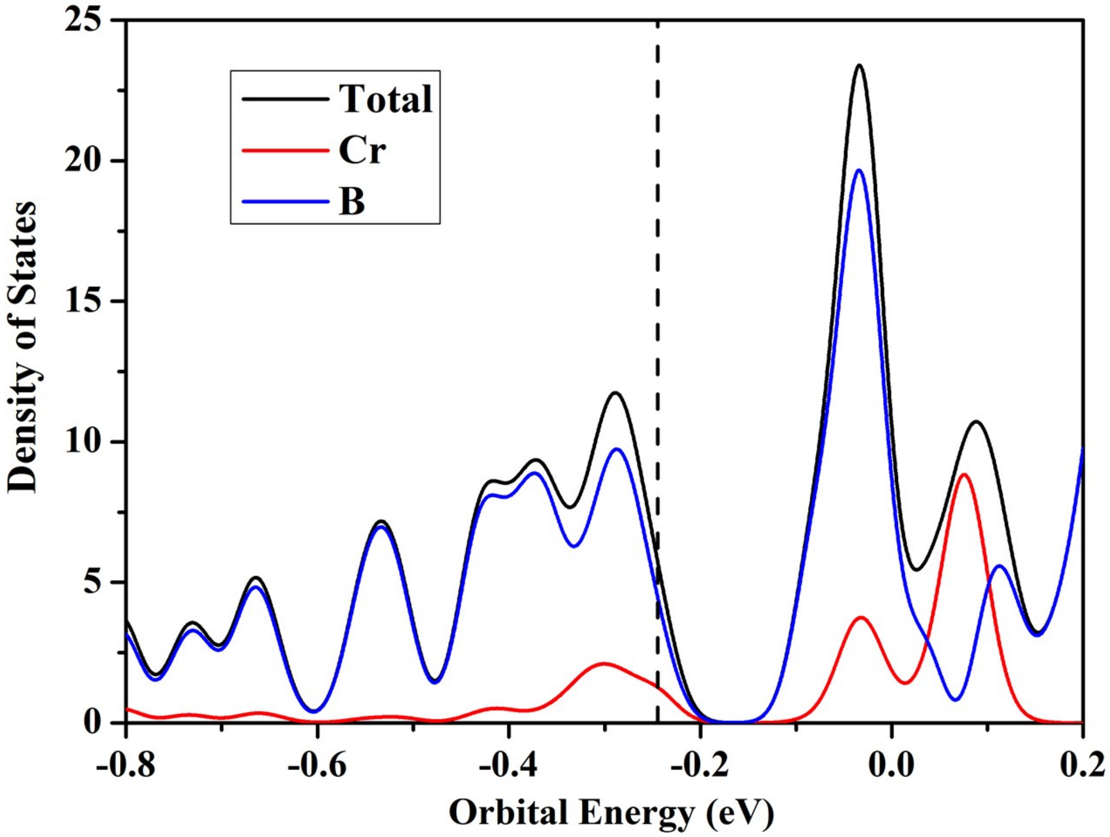

Fig. S12. Total and projected density of states for the endohedral cage-like CrB_{20} cluster. The dashed line highlights the position of HOMO.

Table S1. Cartesian coordinates of the lowest-energy structure of CrB_8

Cr	7.455200	7.685000	8.303500
B	6.400700	6.288800	6.501800
B	9.099400	8.465000	6.572800
B	9.107500	6.921800	6.573000
B	6.386100	9.069500	6.501500
B	7.512100	7.685000	6.136500
B	7.888300	9.420800	6.541000
B	7.906500	5.953300	6.541400
B	5.724000	7.675600	6.484000

Table S2. Cartesian coordinates of the lowest-energy structure of CrB_{10}

Cr	8.645702	7.211105	9.087777
B	6.864284	5.502427	8.372888
B	9.020988	6.792001	6.580219
B	6.223682	8.009082	9.405405
B	7.034672	8.899758	8.364290
B	6.140807	6.438990	9.408009
B	8.380391	9.298630	7.612701
B	6.573325	7.140929	7.972259
B	9.297176	8.326311	6.784362
B	7.732423	7.833989	7.008769
B	7.780659	5.980770	7.161953

Table S3. Cartesian coordinates of the lowest-energy structure of CrB_{12}

Cr	7.408980	12.00170	8.925550
B	9.988865	11.74960	8.455016
B	6.096917	13.38786	7.367074
B	6.357480	10.60534	6.954414
B	5.577452	11.92594	7.033333
B	8.845510	11.98716	7.179896
B	7.263334	12.05038	6.669873
B	9.479504	13.25263	8.456912
B	8.000589	13.41764	7.265269
B	7.903353	10.32969	7.184238
B	8.413115	14.41734	8.614398
B	6.952396	14.47569	8.143725
B	9.308192	10.48736	7.905393

Table S4. Cartesian coordinates of the lowest-energy structure of CrB_{14}

Cr	8.128003	9.281546	9.685618
B	7.890778	10.49034	7.634534
B	7.618474	11.34589	8.887793
B	9.237603	7.786292	8.152818
B	10.11191	9.055685	8.813913
B	9.305986	10.67073	8.418975
B	7.896232	10.49345	11.73542
B	7.620565	11.34703	10.48153
B	9.241713	7.788647	11.21756
B	10.11438	9.056974	10.55232
B	9.309338	10.67265	10.94699
B	8.751063	9.127525	7.529478
B	8.756863	9.130798	11.84024
B	9.560383	7.535482	9.684950
B	10.28896	10.43102	9.681860

Table S5. Cartesian coordinates of the lowest-energy structure of CrB_{16}

Cr	9.624430	9.754655	9.617720
B	8.821323	8.619560	11.23690
B	10.45231	8.033580	10.94501
B	10.00375	9.545160	11.77099
B	8.919185	9.365770	7.598717
B	10.41296	11.815044	10.07717
B	7.805689	8.478948	9.995451
B	11.42911	9.306202	10.74312
B	9.307754	11.74265	8.908493
B	9.316485	7.518392	9.953526
B	10.80018	10.86572	11.30281
B	9.742898	10.75500	7.521438
B	7.520514	9.350567	8.686934
B	11.60741	10.54971	9.771393
B	8.164130	10.78749	8.342902
B	8.759641	8.108065	8.555354
B	10.934278	11.22797	8.467739

Table S6. Cartesian coordinates of the lowest-energy structure of CrB_{18}

Cr	9.484292	9.598221	9.596623
B	8.976945	10.45209	7.577232
B	8.521071	9.643048	11.75060
B	9.730086	11.54307	8.491449
B	7.573799	10.27316	8.942234
B	8.074629	9.163772	7.808622
B	8.921591	7.755554	8.350101
B	9.767626	8.876038	7.511701
B	10.74662	10.07216	7.990947
B	9.447378	8.251537	11.23415
B	10.24903	9.594576	11.67276
B	10.99756	10.76240	10.86963
B	9.232151	11.02627	11.29130
B	8.329586	11.36782	9.859221
B	7.770250	8.555643	10.83934
B	11.20853	10.96310	9.260464
B	9.916308	11.83123	10.04379
B	7.537472	8.571655	9.234528
B	8.814145	7.516334	9.931367

Table S7. Cartesian coordinates of the lowest-energy structure of CrB_{20}

Cr	9.584947	9.635535	9.630617
B	9.753406	9.785738	11.81456
B	8.983707	9.789686	7.524600
B	7.520008	9.070878	10.15126
B	8.580328	7.691453	9.809075
B	9.959386	7.519548	8.889377
B	11.82478	9.913027	9.901116
B	11.02234	11.27526	9.374230
B	9.342607	11.82279	9.525436
B	10.36000	8.703804	7.790768
B	11.32468	9.941580	8.313984
B	10.03502	11.13862	8.094803
B	8.391316	11.10006	8.366722
B	7.637407	9.634861	8.600584
B	8.708270	8.306085	8.119140
B	10.89440	10.66063	11.06416
B	9.225208	11.25880	11.07611
B	8.164302	10.00949	11.36525
B	8.702090	8.436803	11.24436
B	10.15248	7.706890	10.53178
B	11.11716	8.944666	11.05500

Table S8. Cartesian coordinates of the lowest-energy structure of CrB_{22}

Cr	9.633957	9.811267	9.887219
B	11.00359	10.00034	7.899784
B	8.709324	10.84326	11.91010
B	8.834966	7.548270	9.876499
B	10.54646	7.571715	9.570152
B	9.913372	11.95653	9.268650
B	8.414387	11.40262	8.865994
B	11.54533	8.549652	10.60443
B	9.504776	9.446677	7.497333
B	7.961305	11.31284	10.53267
B	10.01762	7.896601	11.09237
B	11.75764	9.477294	9.263734
B	7.393579	10.16376	9.518860
B	10.60263	8.418262	8.196409
B	9.524230	11.89029	10.95246
B	10.79828	9.224353	11.84426
B	11.04902	11.51433	10.50062
B	8.360643	8.323800	11.18953
B	11.37080	11.16110	8.982868
B	8.073254	9.942856	8.097206
B	8.724533	8.356861	8.449228
B	10.36217	10.76718	11.93347
B	7.663247	9.770044	11.20857
